# Stimulation of Inositol 1,4,5-Trisphosphate (IP_3_) Receptor Subtypes by Analogues of IP_3_

**DOI:** 10.1371/journal.pone.0054877

**Published:** 2013-01-25

**Authors:** Huma Saleem, Stephen C. Tovey, Taufiq Rahman, Andrew M. Riley, Barry V. L. Potter, Colin W. Taylor

**Affiliations:** 1 Department of Pharmacology, University of Cambridge, Cambridge, United Kingdom; 2 Wolfson Laboratory of Medicinal Chemistry, Department of Pharmacy and Pharmacology, University of Bath, Bath, United Kingdom; Medical School of Hannover, United States of America

## Abstract

Most animal cells express mixtures of the three subtypes of inositol 1,4,5-trisphosphate receptor (IP_3_R) encoded by vertebrate genomes. Activation of each subtype by different agonists has not hitherto been examined in cells expressing defined homogenous populations of IP_3_R. Here we measure Ca^2+^ release evoked by synthetic analogues of IP_3_ using a Ca^2+^ indicator within the lumen of the endoplasmic reticulum of permeabilized DT40 cells stably expressing single subtypes of mammalian IP_3_R. Phosphorylation of (1,4,5)IP_3_ to (1,3,4,5)IP_4_ reduced potency by ∼100-fold. Relative to (1,4,5)IP_3_, the potencies of IP_3_ analogues modified at the 1-position (malachite green (1,4,5)IP_3_), 2-position (2-deoxy(1,4,5)IP_3_) or 3-position (3-deoxy(1,4,5)IP_3_, (1,3,4,5)IP_4_) were similar for each IP_3_R subtype. The potency of an analogue, (1,4,6)IP_3_, in which the orientations of the 2- and 3-hydroxyl groups were inverted, was also reduced similarly for all three IP_3_R subtypes. Most analogues of IP_3_ interact similarly with the three IP_3_R subtypes, but the decrease in potency accompanying removal of the 1-phosphate from (1,4,5)IP_3_ was least for IP_3_R3. Addition of a large chromophore (malachite green) to the 1-phosphate of (1,4,5)IP_3_ only modestly reduced potency suggesting that similar analogues could be used to measure (1,4,5)IP_3_ binding optically. These data provide the first structure-activity analyses of key IP_3_ analogues using homogenous populations of each mammalian IP_3_R subtype. They demonstrate broadly similar structure-activity relationships for all mammalian IP_3_R subtypes and establish the potential utility of (1,4,5)IP_3_ analogues with chromophores attached to the 1-position.

## Introduction

**Figure 1:**
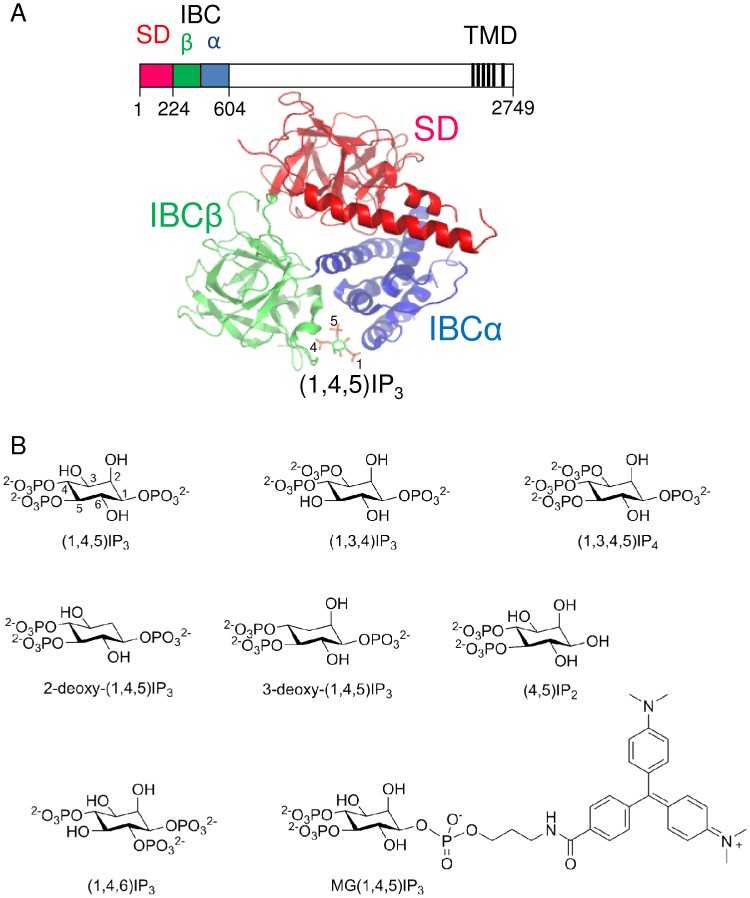
Structure of the N-terminal of the IP_3_ receptor and structures of the ligands used. (A) Key regions of a single IP_3_R subunit (numbering for rat IP_3_R1) are shown highlighting N-terminal domains and the six C-terminal transmembrane domains (TMD) that form the pore. A high-resolution structure of the N-terminal (NT, residues 1–604) with (1,4,5)IP_3_ bound is shown (Protein Data Bank, 3UJO). The NT comprises the suppressor domain (SD) and IP_3_-binding core (IBC). The essential 4- and 5-phosphate groups of (1,4,5)IP_3_ interact with residues in the β-domain and α-domain of the IBC, respectively. (B) Structures of the ligands used.

Most animal cells express inositol 1,4,5-trisphosphate receptors (IP_3_R), which fulfil an essential role in linking the many cell-surface receptors that stimulate IP_3_ formation to release of Ca^2+^ from the endoplasmic reticulum [1]. Vertebrates have genes for three IP_3_R subunits, while invertebrates have only a single IP_3_R gene. All functional IP_3_R are tetrameric assemblies of these subunits. The similar primary sequences of the IP_3_R subunits suggest that all IP_3_R are likely to share similar structures, although we presently have only a limited understanding of the structure of the entire IP_3_R [1], [2]. Each subunit has an N-terminal region to which IP_3_ binds. This region comprises the N-terminal suppressor domain (SD, residues 1–223) and the IP_3_-binding core (IBC, residues 224–604 in IP_3_R1, Figure 1A), which is alone sufficient to bind IP_3_ with appropriate selectivity [3]. The SD both modulates the affinity of the IBC for agonists and provides an essential link between IP_3_ binding and opening of the pore [4], [5], [6], [7]. A large cytoplasmic region separates the N-terminal from the six transmembrane domains. The last pair of these, together with the intervening luminal loop, form the Ca^2+^-permeable pore [8] (Figure 1A). Each subunit terminates in a short C-terminal tail, which has also been implicated in the regulation of gating [9]. The diversity provided by three genes is further increased by multiple splice variants of at least two of the three IP_3_R subtypes (IP_3_R1 and IP_3_R2), by formation of homo- or hetero-tetrameric assemblies of IP_3_R subunits, by association with an enormous diversity of modulatory proteins and by post-translational modifications [10]. At present, we have only a limited understanding of the functional significance of this complexity for IP_3_-evoked Ca^2+^ signals in native tissues.

The broadly similar structures of the three IP_3_R subunits are matched by many shared functional properties, most notably co-regulation of all IP_3_R by IP_3_ and Ca^2+^ [10], [11]. Nevertheless, there are differences in the patterns of expression of IP_3_R in different tissues [12], [13], in their subcellular distributions [14], [15], sensitivities to IP_3_ [16], modulation by accessory proteins and additional signals [17], [18], [19], and in the functional consequences of IP_3_R ablation [20], [21]. Heterogeneous populations of IP_3_R in most cells make it difficult to establish clearly the characteristics of each IP_3_R subtype and to define their functional roles. A better knowledge of the ligand recognition properties of the three IP_3_R3 subtypes is needed if ligands selective for IP_3_R subtypes are to be developed to help resolve these problems. All known high-affinity agonists of IP_3_R retain structures equivalent to the 4,5-bisphosphate and 6-hydroxyl groups of (1,4,5)IP_3_ (Figure 1B) [22]. The only exception is a low-affinity analogue of adenophostin A (3″-dephospho-adenophostin A) in which interactions between the adenine moiety and IP_3_R appear partially to compensate for loss of a phosphate (equivalent to the 5-phosphate of (1,4,5)IP_3_) within the critical bisphosphate moiety [23]. Here we use a selection of synthetic analogues of IP_3_ that preserve the key structures of the high-affinity agonists to assess their activity at each IP_3_R subtype.

## Materials and Methods

### Materials

Thapsigargin was from Alomone Laboratories (Jerusalem, Israel). The structures of the ligands used and their abbreviations are shown in Figure 1B. (1,4,5)IP_3_ was from Alexis Biochemicals (Nottingham, U.K.). 3-deoxy(1,4,5)IP_3_, (1,3,4)IP_3_ and (1,3,4,5)IP_4_ were from Calbiochem (Nottingham, U.K.). (1,4,6)IP_3_ from both Alexis Biochemicals and synthesized as reported previously [24] was used. Malachite green IP_3_ (MG(1,4,5)IP_3_) was synthesized using the methods described by Inoue *et al*. [25]. (4,5)IP_2_ [26], 2-deoxy(1,4,5)IP_3_ [27] and synthetic (1,3,4,5)IP_4_ [28] were synthesized as previously described. All synthesized ligands were purified by ion-exchange chromatography, fully characterized by the usual spectroscopic methods and accurately quantified by total phosphate assay. ^3^H-IP_3_ (185 Bq/mmol) was from PerkinElmer (Bucks, U.K.).

Anti-peptide antisera to peptides conserved in all mammalian IP_3_R subtypes (AbC, residues 62–75 of rat IP_3_R1) or unique to IP_3_R1 (Ab1, residues 2724–2739) or IP_3_R2 (Ab2, residues 2685–2701) were described previously [29]. A monoclonal antibody that recognizes N-terminal residues (22–230) of IP_3_R3 (Ab3) was from BD Transduction Laboratories (Oxford, U.K.). Anti-β-actin antibody was from AbCam (Cambridge, U.K.). Donkey secondary antibodies (anti-rabbit or anti-mouse) conjugated to horseradish peroxidase were from Santa Cruz Biotechnology (Santa Cruz, CA, U.S.A.). Sources of other materials are provided in earlier publications [30], [31].

### Cell Culture

DT40 cells lacking genes for all three IP_3_R subtypes (DT40-KO cells) [32] and the same cells stably expressing rat IP_3_R1 (GenBank accession number GQ233032.1) [33], mouse IP_3_R2 (GU980658.1) [31] or rat IP_3_R3 (GQ233031.1) [34] were cultured in RPMI 1640 medium supplemented with 10% foetal bovine serum, 1% heat-inactivated chicken serum, 2 mM glutamine and 50 µM 2-mercaptoethanol at 37°C in 95% air and 5% CO_2_. Cells were passaged every 2 days when they reached a density of ∼1.5×10^6^ cells/mL.

### Immunoblotting

Cells (∼7×10^7^) were centrifuged (650 *g*, 5 min), resuspended in Hepes-buffered saline (HBS), re-centrifuged, and the pellet was solubilized in 1 mL of medium containing 140 mM NaCl, 5 mM NaF, 10 mM Tris, 1 mM Na_4_P_2_O_7_, 0.4 mM Na_3_VO_4_, 1% Triton X-100 and a protease inhibitor tablet (1 tablet/10 mL, Roche Diagnostics, Mannheim, Germany). HBS had the following composition: 135 mM NaCl, 5.9 mM KCl, 1.2 mM MgCl_2_, 1.5 mM CaCl_2_, 11.6 mM Hepes, 11.5 mM glucose, pH 7.3. The solubilized cells were sonicated on ice (Trans Scientific 1420 sonicator, 50–60 Hz, 3×10 s), incubated with gentle rotation for 1 h at 2°C and then centrifuged (6000 *g*, 10 min). A sample of the supernatant (13 µL) was mixed with dl-dithiothreitol (2 µL, 100 mM) and NuPAGE LDS sample buffer (5 µL, Invitrogen, Paisley, U.K.). After heating (70°C, 10 min), 20-µL samples were loaded onto NuPAGE 3–8% Tris acetate gels for SDS-PAGE using Novex Tris acetate buffer (Invitrogen). Broad range spectrum marker and MagicMark molecular weight markers (Invitrogen) were used to monitor protein migration during SDS-PAGE and for calibration, respectively. After transfer to a PVDF membrane using an iBlot dry-blotting system (Invitrogen), the membrane was blocked by incubation (1 h) with Tris-buffered saline (TBS) containing 5% non-fat dry milk. It was then incubated with primary antiserum (1∶1000 and 1∶10,000 for IP_3_R and β-actin antibodies, respectively) in TBS containing 2.5% w/v BSA for 12 h. TBS had the following composition: 140 mM NaCl, 20 mM Tris, 0.1% v/v Tween, pH 7.6. After incubation with primary antibodies, the blots were washed in TBS (3×5 min), incubated with horseradish peroxidase-conjugated secondary antibodies (1∶1000, donkey anti-rabbit or donkey anti-mouse) for 1 h in TBS supplemented with 2.5% BSA, and washed again (3×5 min). Bands were detected using Supersignal West Pico chemiluminescent substrate (ThermoScientific, Rockford, IL, U.S.A.) and quantified using GeneTools software (Syngene, Frederick, MD, U.S.A.).

### Measurement of IP_3_-evoked Ca^2+^ Release

A low-affinity Ca^2+^ indicator (Mag fluo-4) trapped within the ER lumen was used to measure IP_3_-evoked Ca^2+^ release from permeabilized DT40 cells stably expressing mammalian IP_3_R [35]. Cells (50 mL, 10^6^ cells/mL) were loaded with Mag fluo-4 AM (20 µM) in HBS supplemented with pluronic F127 (0.02% w/v) for 1 h at 20°C in the dark with gentle shaking. The cells were centrifuged (650 *g*, 5 min), resuspended in Ca^2+^-free cytosol like medium (CLM) and permeabilized by addition of saponin (10 µg/mL, ∼4 min, 37°C). Ca^2+^-free CLM had the following composition: 140 mM KCl, 2 mM NaCl, 1 mM EGTA, 2 mM MgCl_2_, 20 mM Pipes, pH 7. After washing (650 *g*, 2 min), permeabilized cells were resuspended in CLM without Mg^2+^, but supplemented with CaCl_2_ (375 µM) to give a final free [Ca^2+^] of ∼220 nM (after addition of 1.5 mM MgATP) and with carbonyl cyanide 4-trifluoromethoxy-phenyl hydrazone (FCCP, 10 µM) to inhibit mitochondrial Ca^2+^ uptake. Cells were distributed (5×10^5^ cells/well) into half area 96-well, black-walled, poly-lysine-coated plates and centrifuged (300 *g*, 2 min). Fluorescence (excitation and emission wavelengths of 485 nm and 520 nm, respectively) was recorded at 1-s intervals using a FlexStation™ 3 fluorescence plate reader (Molecular Devices, Berkshire, U.K.) at 20°C. Addition of MgATP (1.5 mM) allowed Ca^2+^ uptake into the intracellular stores, and after 150 s, IP_3_ (or its analogues) was added together with thapsigargin (1 µM) to prevent further Ca^2+^ uptake. IP_3_-evoked Ca^2+^ release is expressed as a fraction of that released by addition of ionomycin (1 µM) [35].

### ^3^H-IP_3_ Binding to Native Type 1 IP_3_ Receptors

Mouse cerebellar membranes (5 mg protein) in a final volume of 500 µL of CLM with a free [Ca^2+^] of 220 nM were incubated with ^3^H-IP_3_ (1.5 nM) and appropriate concentrations of competing ligand at 4°C [4]. After 5 min, during which equilibrium was attained, the reactions were terminated by centrifugation (2000 *g*). The supernatant was removed and the pellet was washed (500 µL CLM) and then solubilized (200 µL CLM with 1% v/v Triton-X-100) before determining its radioactivity using Ecoscint A scintillation cocktail (National Diagnostics, Atlanta, GA, USA, 1 mL). Total ^3^H-IP_3_ binding was ∼3000 disintegrations/min (d.p.m.) and non-specific binding was ∼300 d.p.m. (determined by addition of 1 µM IP_3_ or by extrapolation of IP_3_ competition curves to infinite IP_3_ concentration). Results were fitted to Hill equations (GraphPad Prism, version 5, GraphPad Software Inc., CA, U.S.A.) from which half-maximal inhibitory concentration (IC_50_) and thereby K_D_ values were calculated.

### Statistical Analysis

Concentration-effect relationships were fitted to Hill equations using Graph Pad Prism, from which Hill coefficients (h), the fraction of the intracellular Ca^2+^ stores released by maximally effective concentrations of agonist, and pEC_50_ values were calculated. For clarity, some results are reported as EC_50_ values, but all statistical comparisons used pEC_50_ values. Each experiment included an analysis of the effects of (1,4,5)IP_3_ to allow paired comparisons of pEC_50_ values for IP_3_ and each analogue (ΔpEC_50_). Results are expressed as means ± SEM for n independent experiments, with each experiment performed in triplicate. Statistical comparisons used paired Student’s t test or ANOVA followed by Bonferroni test, with *P*<0.05 is considered significant.

### Homology Modelling and Ligand Docking

Sequence alignments were performed using MUSCLE (http://www.ebi.ac.uk/Tools/msa/muscle/). Structural homology models were built in Modeller 9.10 [36] using templates of crystal structures of the IBC (Protein Data Bank: 1N4K) and SD (Protein Data Bank: 1XZZ). The geometric qualities of the models were evaluated with the Molprobity server [37]. Finally, the N-terminal regions of mouse IP_3_R2 and rat IP_3_R3 comprising the modelled SD and IBC structures of each were reconstructed by aligning the individual domains against cognate regions of the NT of rat IP_3_R1 (Protein Data Bank: 3UJ4) [7] using UCSF Chimera 1.6.1 [38].

We used docking of IP_3_ analogues into a rigid IBC and subsequent superposition of the structure onto a model of the entire IP_3_R solely to assess whether full-length IP_3_R was likely to bind the analogues without steric clashes. Docking of IP_3_ analogues was performed using the IBC (Protein Data Bank: 1N4K) [3] and AutoDock Vina 1.1.2 [39]. Prior to docking, bound (1,4,5)IP_3_ and all water molecules (except those within 5 Å of the IP_3_-binding site) were removed. The search space comprised a grid of 20×20×20 points, each separated by 0.375 Å, around the IP_3_-binding site. Ligands (except (1,4,5)IP_3_) were drawn and energy-minimized with MM2 force field using ChemBioOffice 2008 (http://www.cambridgesoft.com). Polar hydrogens and the Gasteiger partial atomic charges were then added to the protein and ligands using AutoDockTools (http://autodock.scripps.edu/resources/adt), and the prepared structures were used as input files for docking. Only the best pose is considered for each ligand. For MG(1,4,5)IP_3_, the ligand was superposed onto (1,4,5)IP_3_ within the NT monomer (Protein Data Bank: 3UJ0A) and the complex (with (1,4,5)IP_3_-removed) was energy-minimized using MMFF94 Forcefield [40]. PyMol was used to present docked structures (http://pymol.sourceforge.net/).

## Results

### Expression of Mammalian IP_3_ Receptor Subtypes in DT40 Cells

**Figure 2:**
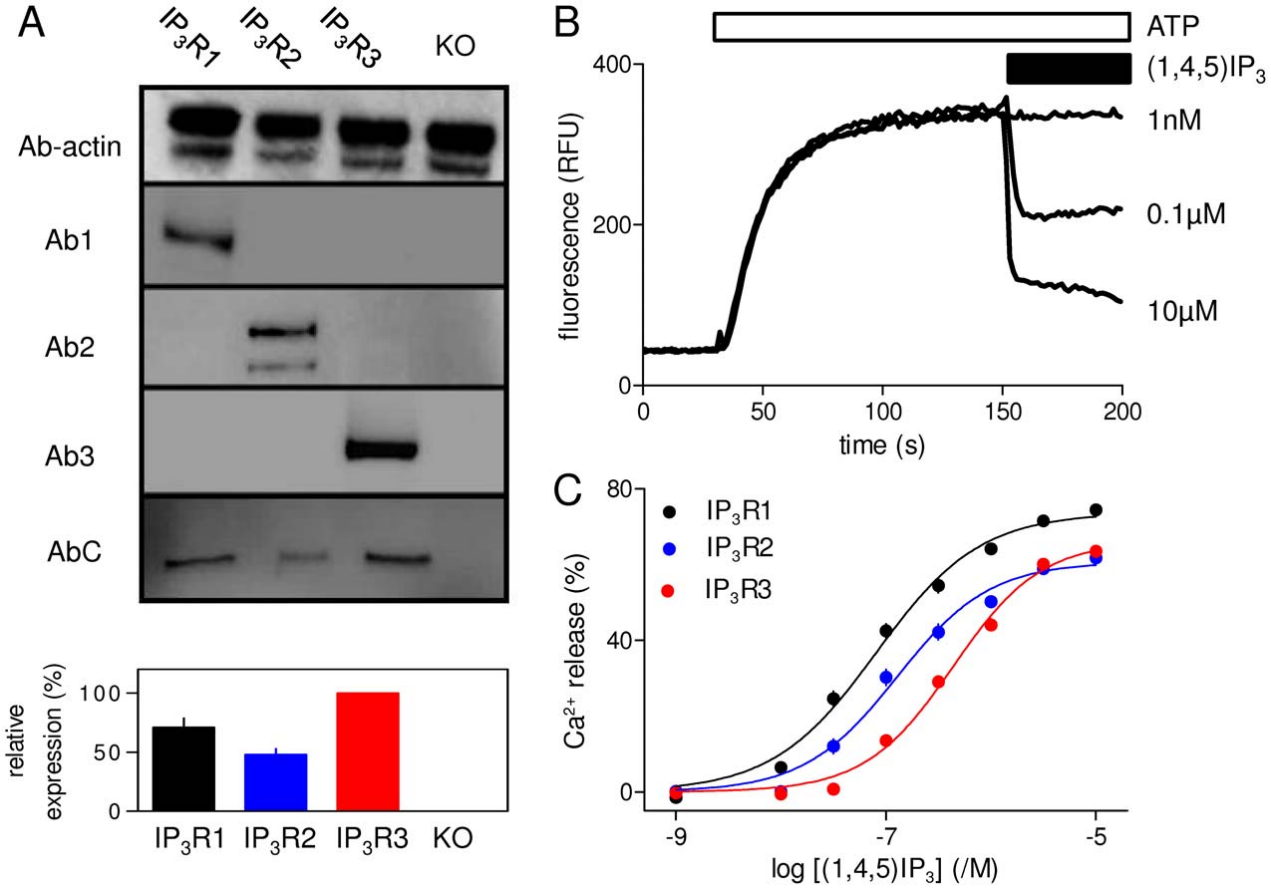
Functional expression of recombinant IP_3_ receptor subtypes in DT40 cells. (A) Western blots from DT40 cells stably expressing each of the IP_3_R subtypes. Each lane was loaded with lysate derived from ∼1.4×10^6^ cells stably expressing IP_3_R1, IP_3_R2 or IP_3_R3, or from DT40-KO cells (KO). The antisera used are selective for IP_3_R1-3 (Ab1-3) or they interact equally with all three IP_3_R subtypes (AbC) [29] (see Materials). An antiserum to β-actin was used to confirm the equivalent loading of lanes. Quantitative analysis of AbC immunoreactivity from 3 similar gels was used to establish expression levels of the three IP_3_R subtypes relative to IP_3_R3 (means ± SEM, n = 3) (lower panel). (B) A luminal Ca^2+^ indicator was used to record Ca^2+^ uptake into the intracellular stores of permeabilized DT40 cells expressing IP_3_R1 after addition of MgATP. Subsequent addition of (1,4,5)IP_3_ (at the concentrations shown) with thapsigargin (1 µM) allowed (1,4,5)IP_3_-evoked Ca^2+^ release to be quantified. The typical experiment shows traces averaged from 3 wells in a single plate. For clarity only a selection of the (1,4,5)IP_3_ concentrations are shown. The same methods were used to quantify the effects of all analogues. (C) Concentration-dependent effects of (1,4,5)IP_3_ on Ca^2+^ release from the intracellular stores of cells expressing IP_3_R1 (black), IP_3_R2 (blue) or IP_3_R3 (red). The same colour-codes are used in all subsequent figures. Results are means ± SEM from the number of independent experiments given in Table 1. Here, and in many subsequent figures, some error bars are smaller than the symbols.

Immunoblotting with IP_3_R subtype-selective antisera (Ab1-3) established that each of the stable DT40 cell lines specifically expressed only a single IP_3_R subtype (Figure 2A). An antiserum that recognizes a peptide sequence present in all mammalian IP_3_R subtypes (AbC) [29] was used to quantify relative levels of IP_3_R expression in the three cell lines (Figure 2A). The results demonstrate that, relative to IP_3_R3 (100%), levels of expression of IP_3_R1 and IP_3_R2 were 71±8% and 48±5%, respectively (Figure 2A). Because the density of IP_3_R may affect the sensitivity of intracellular Ca^2+^ stores to IP_3_ [6] and it is impracticable to generate cell lines expressing identical levels of each IP_3_R subtype, comparisons of the relative potencies of IP_3_ analogues for each IP_3_R subtype are expressed relative to the potency of (1,4,5)IP_3_ in the same cell line 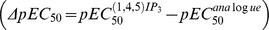 (see Methods).

### IP_3_-evoked Ca^2+^ Release by Subtypes of IP_3_ Receptor

A low-affinity luminal Ca^2+^ indicator was used to report the free Ca^2+^ concentration within the endoplasmic reticulum of permeabilized DT40 cells (Figure 2B). IP_3_ failed to evoke Ca^2+^ release in DT40-KO cells, which lack IP_3_R (not shown) [34], but it was effective in DT40 cells stably expressing each of the three IP_3_R subtypes (Figure 2B and C) [31]. The results demonstrate that in each cell line, IP_3_ caused a concentration-dependent release of 60–75% of the intracellular Ca^2+^ stores, with Hill coefficients (h) of about 1, and pEC_50_ values of 7.06±0.05, 6.84±0.06 and 6.38±0.05 for IP_3_R1, IP_3_R2 and IP_3_R3, respectively (Table 1 and Figure 2C). Notwithstanding the moderate differences in IP_3_R expression in the different DT40 cell lines (Figure 2A), the relative sensitivities of IP_3_R1-3 to IP_3_ in these assays (IP_3_R1∼IP_3_R2>IP_3_R3) are consistent with a general consensus that IP_3_R3 is the least sensitive of the IP_3_R subtypes [16].

**Table 1:** Effects of IP_3_ analogues on Ca^2+^ release by subtypes of IP_3_ receptor.

	IP_3_R1					IP_3_R2					IP_3_R3				
	EC_50_ (nM)	pEC_50_	h	Ca^2+^release (%)	n	EC_50_ (nM)	pEC_50_	h	Ca^2+^release (%)	n	EC_50_ (nM)	pEC_50_	h	Ca^2+^release (%)	n
(1,4,5)IP_3_	87	7.06±0.05	0.99±0.05	75±1	31	145	6.84±0.06	1.26±0.09	61±2	34	417	6.38±0.05	1.26±0.07	64±2	30
(4,5)IP_2_	8128	5.09±0.12	1.11±0.15	69±4	4	6310	5.20±0.20	1.67±0.27	56±4	5	11482	4.94±0.07	1.28±0.03*	83±2	3
(1,3,4,5)IP_4_	11749	4.93±0.05	1.09±0.16	66±6	3	5495	5.26±0.09	2.26±0.18	61±5	3	114815^b^	3.94±0.03	1.08±0.22	36±1^a^	3
2-deoxy(1,4,5)IP_3_	123	6.91±0.15	0.84±0.07	82±3	4	115	6.94±0.11	1.71±0.37	65±2	6	324	6.49±0.02	1.36±0.10	74±3	3
(1,4,6)IP_3_	4365	5.36±0.19	0.98±0.10	81±4	6	12589	4.90±0.16	1.06±0.17	68±2	6	15849	4.80±0.12	2.58±0.61*	46±1	6
3-deoxy(1,4,5)IP_3_	3311	5.48±0.09	1.75±0.24*	61±2	6	5888	5.23±0.07	1.78±0.34	57±6	6	15849	4.80±0.13	2.59±0.68	53±7	6
MG(1,4,5)IP_3_	447	6.35±0.12	0.95±0.10	73±4	9	708	6.15±0.09	1.04±0.08	57±3	9	1738	5.76±0.11	1.18±0.11	64±4	8
(1,3,4)IP_3_	ND	ND	ND	39±8^a^	3	ND	ND	ND	18±6^a^	3	ND	ND	ND	2±1^a^	3

The EC_50_, pEC_50_, Hill coefficient (h) and fraction of the intracellular Ca^2+^ stores released by a maximally effective concentration of each analogue are shown for each IP_3_R subtype. All results (except EC_50_) are shown as means ± SEM from n independent experiments. The (1,3,4,5)IP_4_ was provided by Calbiochem. ND, not determined.

a Ca^2+^ release evoked with 100 µM of the analogue (where the highest attainable concentrations of ligand failed to saturate the response).

b EC_50_ estimated by assuming that maximally effective concentrations of (1,3,4,5)IP_4_ and (1,4,5)IP_3_ release the same fraction of the Ca^2+^ stores.

* Denotes Hill slopes that are significantly different (*P*<0.05) from 1. Statistical comparisons of maximal Ca^2+^ release and pEC_50_ values used paired comparisons (Δmax or ΔpEC_50_), which are presented in Table 2.

### Interactions of IP_3_ Metabolites with IP_3_ Receptor Subtypes

**Figure 3:**
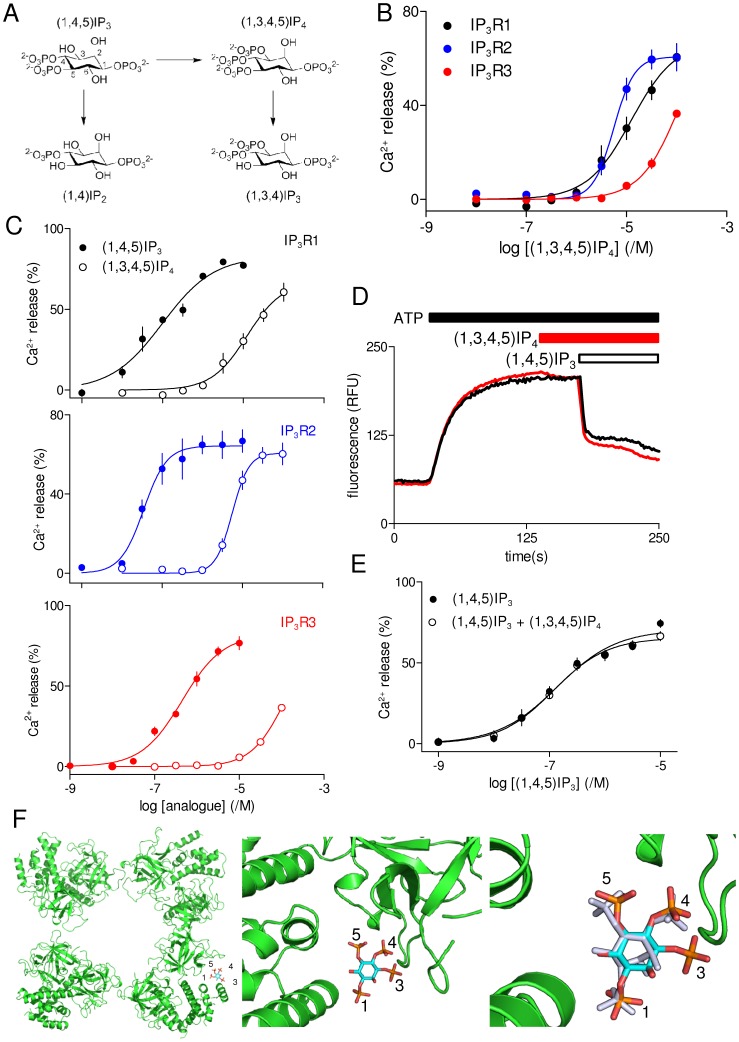
(1,3,4,5)IP_4_ is a low-affinity full agonist of IP_3_ receptors. (A) (1,4,5)IP_3_ can be dephosphorylated to (1,4)IP_2_ or phosphorylated to (1,3,4,5)IP_4_, which can then be dephosphorylated to (1,3,4)IP_3_. (B) Effects of (1,3,4,5)IP_4_ (Calbiochem) on Ca^2+^ release via each of the three IP_3_R subtypes. (C) Paired comparisons of the effects of (1,4,5)IP_3_ and (1,3,4,5)IP_4_ are shown for each IP_3_R subtype. (D) Typical results from DT40-IP_3_R1 cells stimulated with (1,4,5)IP_3_ alone (black trace) or in the presence of (1,3,4,5)IP_4_ (5 µM, added 35 s before IP_3_; red trace). (E) Summary results show the effect of (1,3,4,5)IP_4_ on the concentration-dependent effects of (1,4,5)IP_3_ on Ca^2+^ release. Results (B, C and E) are means ± SEM from the number of independent experiments given in Table 1. (F) (1,3,4,5)IP_4_ docked into the IBC of IP_3_R1 adopts a conformation in which the underlying (1,4,5)IP_3_ scaffold is only slightly distorted from that of (1,4,5)IP_3_ within the crystal structure of the IBC (right panel) [3]. Docking of the NT into a 3D reconstruction of the entire IP_3_R1 [2] has suggested a likely arrangement of the NT within the tetrameric IP_3_R [see 7] (left panel). Within this proposed arrangement, the IBC can still bind (1,3,4,5)IP_4_ without obvious steric clashes.

Inositol 1,3,4,5-tetrakisphosphate ((1,3,4,5)IP_4_) is the immediate product of (1,4,5)IP_3_ phosphorylation by IP_3_ 3-kinase [41] (Figure 3A). Although (1,3,4,5)IP_4_ stimulated Ca^2+^ release via each of the three IP_3_R subtypes (Figure 3B), it was ∼100-fold less potent than IP_3_. Indeed in DT40-IP_3_R3 cells, which are the least sensitive to IP_3_ (Figure 2C), even 100 µM (1,3,4,5)IP_4_ failed to release the entire IP_3_-sensitive Ca^2+^ store (Figures 3B and C, and Table 1). The purity of (1,3,4,5)IP_4_ supplied by Calbiochem is only ∼98%. We were therefore concerned that its effects on Ca^2+^ release might be mediated by contaminating (1,4,5)IP_3_. However, similar experiments using DT40-IP_3_R1 cells and synthetic (1,3,4,5)IP_4_, where the synthesis provides no opportunity for contamination by (1,4,5)IP_3_ [28], established that the two sources of (1,3,4,5)IP_4_ were equipotent. Relative to (1,4,5)IP_3_, ΔpEC_50_ values were 2.09±0.07 and 1.97±0.04 (n = 3) for commercial and synthetic (1,3,4,5)IP_4_, respectively. Synthetic (1,3,4,5)IP_4_ (100 µM) had no significant effect on the Ca^2+^ content of the intracellular stores of permeabilized DT40 cells lacking IP_3_R (DT40-KO cells) (not shown). We conclude that (1,3,4,5)IP_4_ itself stimulates Ca^2+^ release via all IP_3_R, but only at concentrations ∼100-fold higher than with (1,4,5)IP_3_ (Figures 3B and C). The much reduced potency of (1,3,4,5)IP_4_ is consistent with loss of the 3-hydroxyl group reducing potency (Table 1 and see below) and with docking analyses, which suggest that although the 3-phosphate can be accommodated, the orientations of the phosphate groups are slightly distorted relative to (1,4,5)IP_3_ bound to the IBC (Figure 3F).

It has been suggested that (1,3,4,5)IP_4_ potentiates the Ca^2+^ release evoked by (2,4,5)IP_3_ at IP_3_R1 [42], although in other cells there was no evidence for this interaction [43]. We re-examined this phenomenon by first adding (1,3,4,5)IP_4_ to permeabilized DT40-IP_3_R1 cells at a concentration (5 µM) near its threshold for evoking Ca^2+^ release, and then assessed subsequent responses to (1,4,5)IP_3_ (Figure 3D). The results, which show that (1,3,4,5)IP_4_ has no effect on the response to any concentration of (1,4,5)IP_3_ (Figure 3E), provide no support for the suggestion that (1,3,4,5)IP_4_ potentiates IP_3_-evoked Ca^2+^ release [42]. Furthermore, the lack of inhibition of IP_3_-evoked Ca^2+^ release by (1,3,4,5)IP_4_ demonstrates that it is not a partial agonist of IP_3_R. We conclude that (1,3,4,5)IP_4_ is a low-affinity full agonist of all three IP_3_R subtypes.

Dephosphorylation of (1,3,4,5)IP_4_ by IP_3_ 5-phosphatase produces (1,3,4)IP_3_ (Figure 3A), which can accumulate to concentrations considerably exceeding that of (1,4,5)IP_3_ during sustained stimulation [44]. (1,3,4)IP_3_, even at a concentration of 100 µM, failed to stimulate release of the entire (1,4,5)IP_3_-sensitive Ca^2+^ store in any of the three cell lines (Table 1). (1,3,4)IP_3_ is, therefore, at least 1000-fold less potent than (1,4,5)IP_3_. Comparing the fraction of the Ca^2+^ stores released by 100 µM (1,3,4)IP_3_ in cells expressing each of the three IP_3_R subtypes indicates that the response was greatest in DT40-IP_3_R1 cells and smallest in DT40-IP_3_R3 cells (Table 1). The effects of (1,3,4)IP_3_ on the three IP_3_R subtypes (IP_3_R1> IP_3_R2>> IP_3_R3) therefore match the rank order of potency of (1,4,5)IP_3_ (Table 1), consistent perhaps with minor contamination (<0.1%) of the (1,3,4)IP_3_ with (1,4,5)IP_3_ or (1,3,6)IP_3_ accounting for the activity. These results suggest that (1,3,4)IP_3_ is itself unlikely to bind to IP_3_R. This is consistent with a requirement for a vicinal 4,5-bisphosphate structure in all known inositol phosphate ligands of IP_3_R [45]. It is impossible for us to verify this independently by equilibrium competition ^3^H-IP_3_ binding because DT40 cells are the only homogenous source of IP_3_R subtypes presently available to us, but the low density of IP_3_Rs would require impracticably large numbers of cells for binding studies.

### Type 3 IP_3_ Receptors are Slightly Less Sensitive to Loss of the 1-phosphate from (1,4,5)IP_3_

**Figure 4:**
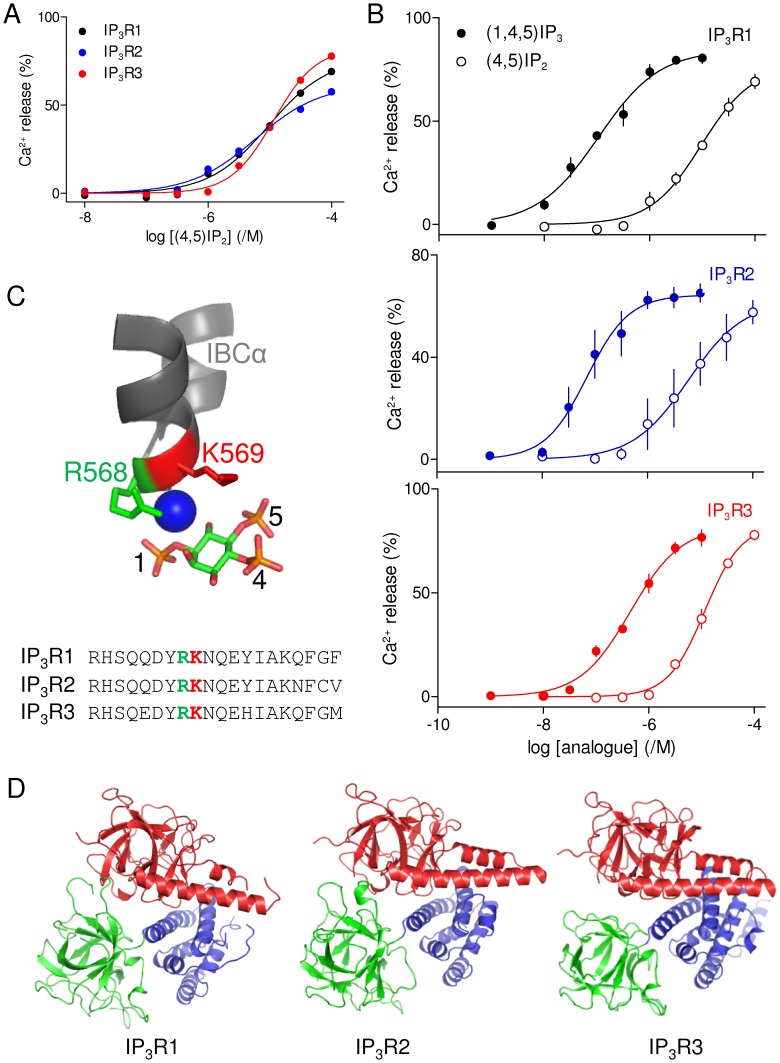
IP_3_ receptor subtypes differ slightly in their requirements for a 1-phosphate group in (1,4,5)IP_3_. (A) Effects of (4,5)IP_2_ on Ca^2+^ release via each IP_3_R subtype. (B) Paired comparisons of the effects of (1,4,5)IP_3_ and (4,5)IP_2_ are shown for each IP_3_R subtype. (C) The 1-phosphate group of (1,4,5)IP_3_ interacts directly with R568 and via water (blue sphere) with K569 within the α-domain of the IBC [3]. The primary sequences of this region (residues 561-580) are similar for each IP_3_R subtype. (D) Structural homology models of the NT of mouse IP_3_R2 and rat IP_3_R3 were built using structures of the SD (Protein Data Bank: 1XZZ) and IBC (Protein Data Bank: 1N4K) from IP_3_R1 and these were then aligned using the published structure of the NT of rat IP_3_R1 (Protein Data Bank: 3UJ0).

Removal of the 1-phosphate from (1,4,5)IP_3_ to give (4,5)IP_2_ caused a similar ∼83-fold decrease in potency for IP_3_R1 and IP_3_R2 (Figures 4A and B, Tables 1 and 2). Contamination of (4,5)IP_2_ with (1,4,5)IP_3_ cannot explain this activity because (4,5)IP_2_ was prepared by total synthesis and purified by ion-exchange chromatography [26]. This is consistent with previous functional analyses of native IP_3_R [45] and with the ability of (4,5)IP_2_ to compete with ^3^H-(1,4,5)IP_3_ for binding to these IP_3_R subtypes heterologously expressed in Sf9 cells [46].

**Table 2:** Relative potencies of IP_3_ analogues at different IP_3_ receptor subtypes.

	IP_3_R1			IP_3_R2			IP_3_R3		
	ΔpEC_50_	Δmax (%)	(1,4,5)IP_3_max (%)	ΔpEC_50_	Δmax (%)	(1,4,5)IP_3_max (%)	ΔpEC_50_	Δmax (%)	(1,4,5)IP_3_max (%)
(4,5)IP_2_	1.92±0.15	13±5	81±3	1.92±0.05	8±3	65±4	1.38±0.06*	−1±2	77±4
2-deoxy(1,4,5)IP_3_	0.09±0.10	−1±2	81±3	0.19±0.14	−3±3	63±4	−0.17±0.08	0±2	77±4
(1,4,6)IP_3_	1.57±0.09	−2±5	76±5	1.67±0.15	−3±6	64±5	1.45±0.09	8±6	55±7
3-deoxy(1,4,5)IP_3_	1.61±0.12	21±2*	80±4	1.64±0.21	12±4*	68±3	1.66±0.17	20±5*	66±3
(1,3,4,5)IP_4_ a	2.09±0.07	17±5^a^	77±1	2.18±0.04	7±0*	67±6	2.38±0.07	ND^a^	77±4
MG(1,4,5)IP_3_	0.69±0.10	4±5	77±3	0.7±0.14	11±3*	64±3	0.64±0.15	4±4	61±3

From paired comparisons with (1,4,5)IP_3_, the relative potency 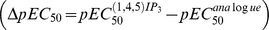 and differences in maximal Ca^2+^ release 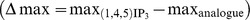 are shown for each analogue at each IP_3_R subtype. Results show means ± SEM, with n provided in Table 1. To allow direct comparison with responses evoked by (1,4,5)IP_3_, maximal Ca^2+^ release evoked by (1,4,5)IP_3_ in experiments paired with the analogues are also shown ((1,4,5)IP_3_ max).

a With (1,3,4,5)IP_4_ it was impossible to attain concentrations (>100 µM) that evoked a maximal response for all IP_3_R subtypes; in these cases the maximal responses were not subject to statistical analysis. The relative potency of (1,3,4,5)IP_4_ at IP_3_R subtypes was estimated by assuming it was a full agonist that released the same fraction of the intracellular Ca^2+^ stores as a maximally effective concentration of (1,4,5)IP_3_ in parallel analyses. ND, not determined.

* Denotes values significantly different (*P*<0.05) from IP_3_R1 (for ΔpEC_50_ values) or from (1,4,5)IP_3_ in paired comparisons with the analogue (for Δmax values).

However, the difference in potency of (1,4,5)IP_3_ and (4,5)IP_2_ at IP_3_R3 was only ∼24-fold (ΔpEC_50_ = 1.38±0.06) (Figure 4B, Table 2). Our results suggest that although removal of the 1-phosphate from (1,4,5)IP_3_ reduces potency at all IP_3_R subtypes, IP_3_R3 is least affected. Within the IBC of IP_3_R1, the 1-phosphate of (1,4,5)IP_3_ interacts directly with R568 and, via water, with K569 [3] (Figure 4C). These residues and their immediate neighbours are conserved in IP_3_R2 and IP_3_R3 (Figure 4C). The only residues known to interact directly with (1,4,5)IP_3_ are within the IBC (Figure 1A) and primary sequences of the IBC are highly conserved between IP_3_R subtypes. It is therefore unsurprising that homology models based on the IBC of IP_3_R1 [3] suggest almost indistinguishable structures for the IBCs from IP_3_R2 and IP_3_R3 (Figure 4D). Furthermore, the IBCs from the three IP_3_R subtypes have the same affinity for (1,4,5)IP_3_ [16]. However, both full-length IP_3_R and the NT from different IP_3_R subtypes differ in their affinities for (1,4,5)IP_3_. These observations demonstrate that interactions between the IBC and other parts of the IP_3_R, notably the suppressor domain (SD, residues 1–223), influence ligand binding [4], [16]. The complexity of these interactions between the IBC and other domains together with the need for only very modest conformational differences between subtypes to account for the small differences in ligand potency make it difficult to define the residues responsible for modulating the interactions between the IBC and the 1-phosphate group of (1,4,5)IP_3_ in IP_3_R3.

### Removal of the 2- and 3-hydroxyl Groups from (1,4,5)IP_3_ or Inverting their Orientations Similarly Affect Interactions with all IP_3_ Receptor Subtypes

**Figure 5:**
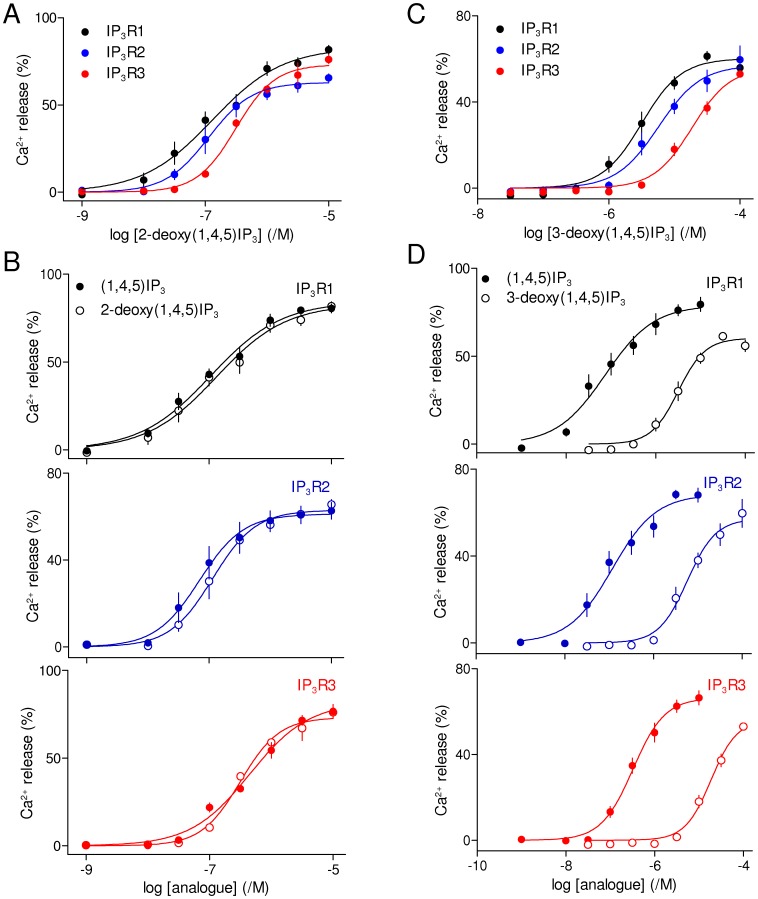
The 2- and 3-hydroxy groups of (1,4,5)IP_3_ interact similarly with each IP_3_ receptor subtype. (A) Effects of 2-deoxy-(1,4,5)IP_3_ on Ca^2+^ release via each IP_3_R subtype. (B) Paired comparisons of the effects of (1,4,5)IP_3_ and 2-deoxy-(1,4,5)IP_3_ are shown for each IP_3_R subtype. (C and D) Similar analyses of 3-deoxy-(1,4,5)IP_3_. Results (A-D) are means ± SEM from the number of independent experiments given in Table 1.

The structure of (1,4,5)IP_3_ bound to the IBC of IP_3_R1 shows the 2-hydroxyl group of (1,4,5)IP_3_ exposed to solvent (Figure 1A) [3] and previous structure-activity studies of native IP_3_R suggested that removal of the 2-hydroxyl moiety from (1,4,5)IP_3_ to give 2-deoxy(1,4,5)IP_3_ minimally affects activity [4], [46]. Our results confirm that observation for all three IP_3_R subtypes: 2-deoxy(1,4,5)IP_3_ and (1,4,5)IP_3_ are equipotent at all three IP_3_R subtypes (Figures 5A and B, Tables 1 and 2).

**Figure 6:**
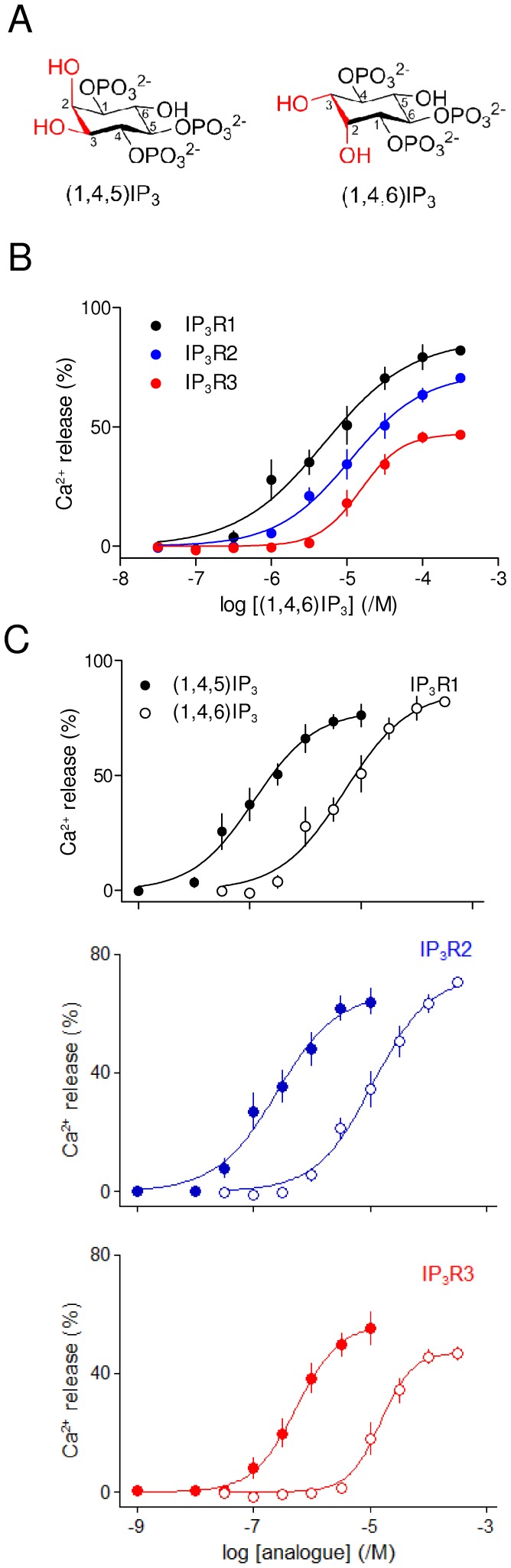
Interactions of (1,4,6)IP_3_ with IP_3_ receptor subtypes. (A) Structures of (1,4,5)IP_3_ and (1,4,6)IP_3_ drawn to show how they differ only in the relative orientations of the 2- and 3-hydroxyl groups of (1,4,5)IP_3_. (B) Effects of (1,4,6)IP_3_ on Ca^2+^ release via each IP_3_R subtype. (C) Paired comparisons of the effects of (1,4,5)IP_3_ and (1,4,6)IP_3_ are shown for each IP_3_R subtype. Results (B and C) are means ± SEM from the number of independent experiments given in Table 1.

Removal of the 3-hydroxyl of (1,4,5)IP_3_ (3-deoxy(1,4,5)IP_3_) caused a ∼40-fold decrease in potency (ΔpEC_50_ ∼1.6) that was similar for all three IP_3_R subtypes (Figures 5C and D, Tables 1 and 2). This decrease is larger than that observed for either Ca^2+^ release from cells expressing largely IP_3_R1 (∼3-fold) [47] or for binding to heterologously expressed IP_3_R1-3 (7- 20-fold) [46].

(1,4,6)IP_3_ is an analogue of (1,4,5)IP_3_ in which the orientations of the 2- and 3-hydroxyl groups are inverted (Figures 1B and 6A). (1,4,6)IP_3_ was less potent than (1,4,5)IP_3_ and the loss of potency was similar for all three IP_3_R subtypes (Figures 6B and C, Tables 1 and 2). The loss of potency probably results from inverting the 3-hydroxyl group from its equatorial position in (1,4,5)IP_3_ to an axial position in (1,4,6)IP_3_ (Figure 6A) because l-*scyllo*(1,2,4)IP_3_, which differs from (1,4,5)IP_3_ only in its inverted orientation of the 2-hydroxyl group, was reported to have the same affinity as (1,4,5)IP_3_ for all three IP_3_R subtypes [46].

### MG-(1,4,5)IP_3_ is a Full Agonist of IP_3_ Receptors with Slightly Lesser Affinity than (1,4,5)IP_3_

MG(1,4,5)IP_3_ in which 4-carboxy-malachite green is attached via an aminopropyl linkage to the 1-phosphate of (1,4,5)IP_3_ (Figure 1B) was originally synthesized to explore its potential as a ligand of IP_3_R that might allow chromophore-assisted laser inactivation (CALI) of IP_3_R [25], [48]. The first study, using surface plasmon resonance to assess binding to an N-terminal fragment of IP_3_R1 (residues 1–885), surprisingly suggested that MG(1,4,5)IP_3_ had ∼170-fold greater affinity than (1,4,5)IP_3_, whereas fluorescein similarly attached to the 1-position of (1,4,5)IP_3_ had no significant effect on affinity [25]. The results were important because they suggested that MG(1,4,5)IP_3_ might be the ligand with the highest known affinity for IP_3_R, and they were unexpected because disrupting interaction of the 1-phosphate group of (1,4,5)IP_3_ with the IP_3_R would be expected to reduce affinity (Figure 4 and Table 1) [45]. A subsequent study of Ca^2+^ release from permeabilized smooth muscle cells concluded that the EC_50_ for MG(1,4,5)IP_3_ was ∼7-fold higher than that for (1,4,5)IP_3_ [25]. The disparity between the reported very high affinity of MG(1,4,5)IP_3_ for the N-terminal of IP_3_R1 and its modest potency in functional assays of smooth muscle has not been explained. We considered two possibilities. MG(1,4,5)IP_3_ may be a high-affinity partial agonist or it may differ massively in its affinity for IP_3_R1 and the endogenous IP_3_R of smooth muscle.

**Figure 7:**
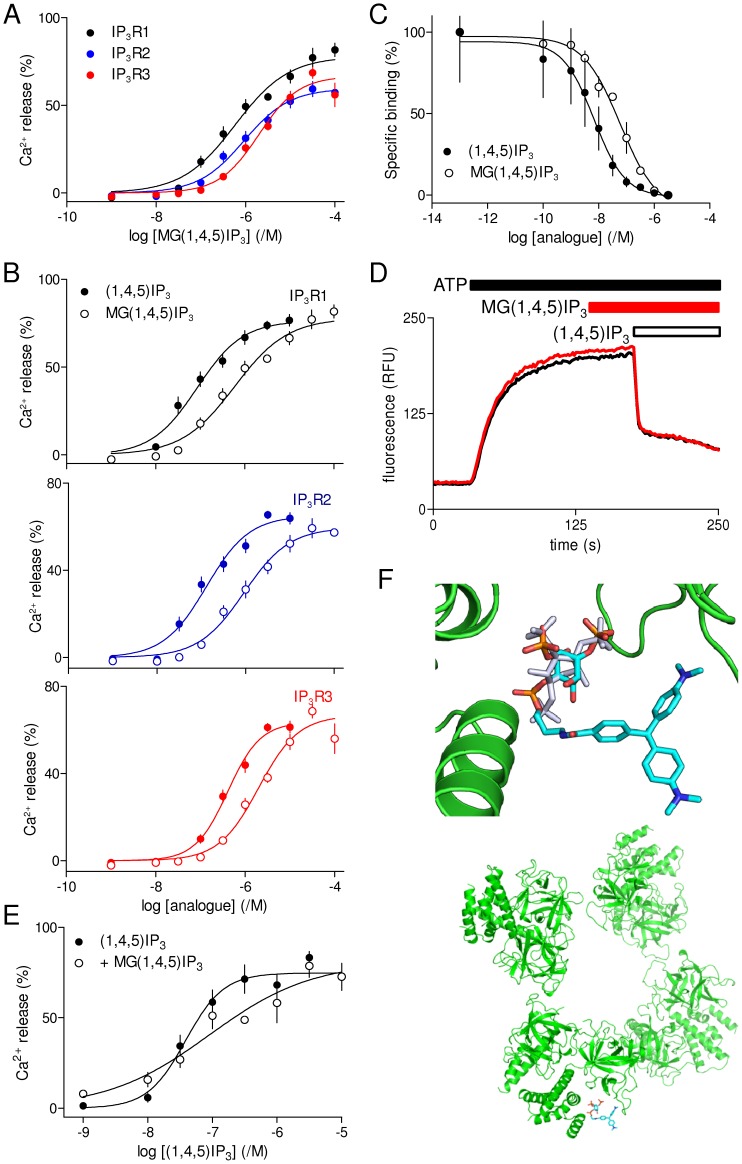
MG(1,4,5)-IP_3_ is a full agonist of all IP_3_ receptor subtypes. (A) Effects of MG(1,4,5)IP_3_ on Ca^2+^ release via each IP_3_R subtype. (B) Paired comparisons of the effects of (1,4,5)IP_3_ and MG(1,4,5)IP_3_ are shown for each IP_3_R subtype. Results (A and B) are means ± S.E.M. from the number of independent experiments given in Table 1. (C) Equilibrium-competition binding of ^3^H-IP_3_ (1.5 nM) to cerebellar membranes in CLM containing 220 nM free [Ca^2+^] in the presence of the indicated concentrations of (1,4,5)IP_3_ or MG(1,4,5)IP_3_. (D) Typical results showing effects of MG(1,4,5)IP_3_ (30 nM, added 35 s before (1,4,5)IP_3_) on Ca^2+^ release from DT40-IP_3_R cells. (E) Summary results (means ± SEM from 3 independent experiments) show concentration-dependent effects of (1,4,5)IP_3_ alone or in the presence of MG(1,4,5)IP_3_ (300 nM). (F) MG(1,4,5)IP_3_ docked into the IBC of IP_3_R1 (top; (1,4,5)IP_3_ shown in gray) shows that it can be accommodated within the likely structure of the tetrameric IP_3_R without steric clashes (bottom).

Our results indicate that MG(1,4,5)IP_3_ is ∼5-fold less potent than (1,4,5)IP_3_ at each IP_3_R subtype (Figures 7A and B, Tables 1 and 2). This is consistent with functional assays of smooth muscle [25] and with our results from native IP_3_R (largely IP_3_R2) in rat hepatocytes, where ΔpEC_50_ was 0.57±0.03 for (1,4,5)IP_3_ (pEC_50_ = 6.81±0.02) and MG(1,4,5)IP_3_ (pEC_50_ = 6.24±0.01) (Taylor CW, unpublished data). These results establish that MG(1,4,5)IP_3_ interacts similarly with all three IP_3_R subtypes and that it is less potent than (1,4,5)IP_3_. In aqueous solution, the triphenylmethane component of MG(1,4,5)IP_3_ exists as a mixture of several inter-converting species, whose relative proportions are sensitive to pH [49]. At pH 7.4, a colourless triphenylmethanol form with a tetrahedral structure is likely to co-exist with the coloured propeller-shaped form shown in Figures 1B and 7F. Because the earlier surface plasma resonance experiments [25], [48] and our analyses were performed at similar pH, we assume that the proportions of the two forms were similar in each analysis.

In equilibrium-competition binding assays in CLM using cerebellar membranes, which predominantly express IP_3_R1 [14], (1,4,5)IP_3_ bound with an affinity that was ∼7-fold greater than that of MG(1,4,5)IP_3_ (ΔpK_D_ = 0.86±0.08, n = 3): pK_D_ = 8.29±0.03 and 7.43±0.08, for (1,4,5)IP_3_ and MG(1,4,5)IP_3_, respectively (Figure 7C and Table 3). The ∼7-fold lesser affinity of MG(1,4,5)IP_3_ for IP_3_R1 relative to (1,4,5)IP_3_ and its similarly reduced potency (∼5-fold, Table 2) are consistent with the large malachite green structure perturbing interaction of the 1-phosphate of (1,4,5)IP_3_ with the IBC, and they suggest that MG(1,4,5)IP_3_ is a full agonist of IP_3_R. The latter conclusion is further substantiated by the results shown in Figures 7D and E. These show that the Ca^2+^ release evoked by (1,4,5)IP_3_ is unaffected by the presence of 300 nM MG(1,4,5)IP_3_, which itself released 34±4% of the Ca^2+^ stores. The pEC_50_ was 7.41±0.09 and 7.01±0.37 (n = 3) for (1,4,5)IP_3_ alone and in the presence of MG(1,4,5)IP_3_, respectively (Figure 7E). A weak partial agonist, by contrast, would be expected, at concentrations sufficient to evoke a response, to occupy enough IP_3_R to shift the concentration-dependence of the response to (1,4,5)IP_3_ to higher concentrations [4]. These results establish that MG(1,4,5)IP_3_ is not a partial agonist of IP_3_R1. We conclude that MG(1,4,5)IP_3_ is a full agonist of IP_3_R with an affinity that is ∼7-fold less than that of (1,4,5)IP_3_.

**Table 3:** Interactions of MG(1,4,5)IP_3_ with type 1 IP_3_ receptors.

	DT40-IP_3_R1 cells			Cerebellum		
	EC_50_ (nM)	pEC_50_ (/M)	Maximal Ca^2+^ release (%)	K_D_ (nM)	pK_D_ (/M)	pK_D_-pEC_50_
(1,4,5)IP_3_	91	7.04±0.1	77±2	5.13	8.29±0.03	1.25±0.29
MG(1,4,5)IP_3_	447	6.35±0.12	73±4	37.2	7.43±0.08	1.08±0.35

Results from functional assays of Ca^2+^ release from DT40-IP_3_R1 cells (Figure 7B) and of equilibrium competition binding to cerebellar membranes (Figure 7C) compare the pEC_50_ and pK_D_ values for (1,4,5)IP_3_ and MG(1,4,5)IP_3_. Results (except EC_50_ and K_D_) show means ± SEM from 3–9 independent experiments. The final column shows pK_D_-pEC_50_ for each ligand.

## Discussion

The primary sequence of the IBC, which is responsible for recognition of (1,4,5)IP_3_ by IP_3_R, is well conserved between IP_3_R subtypes, and in isolation the IBC from each IP_3_R subtype binds IP_3_ with similar affinity [16]. Although there are high-resolution structures of this region for only IP_3_R1 [3], [7], [50], [51], homology modelling suggests that the IBC structures of the three IP_3_R subtypes are very similar (Figure 4D). It is, therefore, unsurprising that structure-activity relationships for the three IP_3_R subtypes, most derived from comparisons of cells expressing mixtures of IP_3_R subtypes, are similar [45], [52], [53]. Nevertheless, residues outside the IBC, notably the SD (Figure 1A), reduce the affinity of the IBC to differing extents for different IP_3_R subtypes [16]. Such differences and the minor differences in the primary sequence of the IBC between IP_3_R subtypes leave open the possibility that it may be possible to develop subtype-selective ligands of IP_3_R. Hitherto, the only systematic comparison of the ligand recognition properties of homogenous populations of IP_3_R subtypes examined only ligand binding to mammalian IP_3_R expressed in insect Sf9 cells [46]. We have now extended the analysis to functional assays of mammalian IP_3_R expressed in a null background, namely DT40 cells in which the genes for all endogenous IP_3_R have been disrupted [32] (Figure 2).

All high-affinity inositol phosphate agonists of IP_3_R have structures equivalent to the 4,5-bisphosphate and 6-hydroxyl groups of (1,4,5)IP_3_ [22], [45], [46], and with the exception of (1,3,4)IP_3_ (which was effectively inactive), all ligands examined herein retain these groups (Figure 1B). Most of the ligands examined were either inactive ((1,3,4)IP_3_) or their potency relative to (1,4,5)IP_3_ suggested a lack of selectivity for IP_3_R subtypes (MG(1,4,5)IP_3_, (1,4,6)IP_3_, (1,3,4,5)IP_4_, 2-deoxy(1,4,5)IP_3_ and 3-deoxy(1,4,5)IP_3_) (Figures 3, 5, 6 and 7, Tables 1 and 2). The potencies of each of these agonists for each IP_3_R subtype are consistent with known structure-activity relationships [45], [46] and with the structure of (1,4,5)IP_3_ bound to the IBC [3] (Figure 1A). Loss of the 2-hydroxyl minimally affects activity, loss of either the 3-hydroxyl or 1-phosphate or inverting the orientation of the 3-hydroxyl causes the potency to decrease substantially, and addition of malachite green to the 1-phosphate of (1,4,5)IP_3_ causes a modest (∼7-fold) decrease in potency (Tables 1 and 2). We conclude that the structure-activity relationships for all three mammalian IP_3_R are broadly similar.

MG(1,4,5)IP_3_ was reported to bind with unexpectedly high affinity to an N-terminal fragment of IP_3_R1 [25], but our results, in line with subsequent analyses of smooth muscle cells from the same group [25], suggest that MG(1,4,5)IP_3_ interacts similarly with all IP_3_R subtypes and that it is a full agonist with an affinity that is ∼7-fold lower than that of (1,4,5)IP_3_ (Figure 7 and Tables 2 and 3). This is consistent with evidence suggesting that even substantial additions to the 1-phosphate of (1,4,5)IP_3_ (eg, fluorescein and 3-aminopropyl) are well-tolerated [25], [48], [54]. It is also consistent with our docking studies, which suggest that even these very large additions to the 1-phosphate moiety of (1,4,5)IP_3_ do not create steric clashes within the tetrameric IP_3_R (Figure 7F).

The necessity of the 4,5-bisphosphate and 6-hydroxyl moieties [55], and the considerable loss of affinity associated with modifying the 3-hydroxyl group of (1,4,5)IP_3_ (Figures 3C, D and 5C, D) restrict opportunities to tag (1,4,5)IP_3_ to modifications of the 1- and 2-positions. Our previous work has shown that modification of the 2-position is compatible with high-affinity binding to IP_3_R, but it reduces efficacy [4], [56]. Our demonstration that MG(1,4,5)IP_3_ is a high-affinity full agonist of IP_3_R (Figure 7) therefore identifies an opportunity to develop fluorescent, or otherwise modified, ligands of IP_3_R that might be expected to come close to mimicking (1,4,5)IP_3_ in their interactions with IP_3_R.

Only one synthetic ligand discriminated modestly between IP_3_R subtypes. (4,5)IP_2_ lacks the 1-phosphate group of the endogenous ligand, (1,4,5)IP_3_ (Figure 1B). In keeping with considerable published evidence [45], [46], this causes a substantial decrease in potency at all IP_3_R subtypes (Figure 4, Tables 1 and 2). The loss of potency is, however, significantly less pronounced (∼24-fold) for IP_3_R3 than for the other IP_3_R subtypes (∼80-fold) (Table 2). The difference is unlikely to be due to residues with the IBC itself because the IBCs of all three IP_3_R subtypes bind (1,4,5)IP_3_ with indistinguishable affinity [16] and the residues that interact with the 1-phosphate group are conserved between IP_3_R (Figure 4C). We instead suggest that subtype-selective interactions of the IBC with other domains, perhaps the SD, subtly modify interaction of the 1-phosphate group of (1,4,5)IP_3_ with two basic residues within the IBC (Figure 4C).

Using homogenous populations of mammalian IP_3_R and a variety of (1,4,5)IP_3_ analogues, we have shown that the three IP_3_R subtypes have very similar ligand recognition properties. However, the decrease in affinity caused by loss of the 1-phosphate group is less for IP_3_R3. Finally, we have shown that MG(1,4,5)IP_3_ is a full agonist of IP_3_R with only modestly reduced affinity, suggesting that attachment of fluorescent tags to the 1-phosphate of (1,4,5)IP_3_ [57] is a feasible strategy for producing modified analogues of (1,4,5)IP_3_ that closely mimic the native messenger.

## References

[1] Taylor CW, Tovey SC (2010) IP_3_ receptors: toward understanding their activation. Cold Spring Harb Persp Biol 2: a004010. 10.1101/cshperspect.a004010 PMC2982166 20980441

[2] Ludtke SJ, Tran TP, Ngo QT, Moiseenkova-Bell VY, Chiu W, et al (2011) Flexible architecture of IP_3_R1 by cryo-EM. Structure 19: 1192–1199. 21827954 10.1016/j.str.2011.05.003 PMC3154621

[3] Bosanac I, Alattia J-R, Mal TK, Chan J, Talarico S, et al (2002) Structure of the inositol 1,4,5-trisphosphate receptor binding core in complex with its ligand. Nature 420: 696–700. 12442173 10.1038/nature01268

[4] Rossi AM, Riley AM, Tovey SC, Rahman T, Dellis O, et al (2009) Synthetic partial agonists reveal key steps in IP_3_ receptor activation. Nat Chem Biol 5: 631–639. 19668195 10.1038/nchembio.195 PMC2869033

[5] Chan J, Yamazaki H, Ishiyama N, Seo MD, Mal TK, et al (2010) Structural studies of inositol 1,4,5-trisphosphate receptor: coupling ligand binding to channel gating. J Biol Chem 285: 36092–36099. 20843799 10.1074/jbc.M110.140160 PMC2975231

[6] Yamazaki H, Chan J, Ikura M, Michikawa T, Mikoshiba K (2010) Tyr-167/Trp-168 in type1/3 inositol 1,4,5-trisphosphate receptor mediates functional coupling between ligand binding and channel opening. J Biol Chem 285: 36081–36091. 20813840 10.1074/jbc.M110.140129 PMC2975230

[7] Seo M-D, Velamakanni S, Ishiyama N, Stathopulos PB, Rossi AM, et al (2012) Structural and functional conservation of key domains in InsP_3_ and ryanodine receptors. Nature 483: 108–112. 22286060 10.1038/nature10751 PMC3378505

[8] Ramos-Franco J, Galvan D, Mignery GA, Fill M (1999) Location of the permeation pathway in the recombinant type-1 inositol 1,4,5-trisphosphate receptor. J Gen Physiol 114: 243–250. 10436000 10.1085/jgp.114.2.243 PMC2230642

[9] Uchida K, Miyauchi H, Furuichi T, Michikawa T, Mikoshiba K (2003) Critical regions for activation gating of the inositol 1,4,5-trisphosphate receptor. J Biol Chem 278: 16551–16560. 12621039 10.1074/jbc.M300646200

[10] Foskett JK, White C, Cheung KH, Mak DO (2007) Inositol trisphosphate receptor Ca^2+^ release channels. Physiol Rev 87: 593–658. 17429043 10.1152/physrev.00035.2006 PMC2901638

[11] Marchant JS, Taylor CW (1997) Cooperative activation of IP_3_ receptors by sequential binding of IP_3_ and Ca^2+^ safeguards against spontaneous activity. Curr Biol 7: 510–518. 9210378 10.1016/s0960-9822(06)00222-3

[12] Fujino I, Yamada N, Miyawaki A, Hasegawa M, Furuichi T, et al (1995) Differential expression of type 2 and type 3 inositol 1,4,5-trisphosphate receptor mRNAs in various mouse tissues: in situ hybridization study. Cell Tissue Res 280: 201–210. 7781020 10.1007/BF00307790

[13] Yamamoto-Hino M, Miyawaki A, Kawano H, Sugiyama T, Furuichi T, et al (1995) Immunohistochemical study of inositol 1,4,5-trisphosphate receptor type 3 in rat central nervous system. Neuroreport 6: 273–276. 7756608 10.1097/00001756-199501000-00012

[14] Taylor CW, Genazzani AA, Morris SA (1999) Expression of inositol trisphosphate receptors. Cell Calcium 26: 237–251. 10668562 10.1054/ceca.1999.0090

[15] Vermassen E, Parys JB, Mauger J-P (2004) Subcellular distribution of the inositol 1,4,5-trisphosphate receptors: functional relevance and molecular determinants. Biol Cell 96: 3–17. 15093123 10.1016/j.biolcel.2003.11.004

[16] Iwai M, Michikawa T, Bosanac I, Ikura M, Mikoshiba K (2007) Molecular basis of the isoform-specific ligand-binding affinity of inositol 1,4,5-trisphosphate receptors. J Biol Chem 282: 12755–12764. 17327232 10.1074/jbc.M609833200

[17] Patterson RL, Boehning D, Snyder SH (2004) Inositol 1,4,5-trisphosphate receptors as signal integrators. Annu Rev Biochem 73: 437–465. 15189149 10.1146/annurev.biochem.73.071403.161303

[18] Ando H, Mizutani A, Matsu-ura T, Mikoshiba K (2003) IRBIT, a novel inositol 1,4,5-trisphosphate (IP_3_) receptor-binding protein, is released from the IP_3_ receptor upon IP_3_ binding to the receptor. J Biol Chem 278: 10602–10612. 12525476 10.1074/jbc.M210119200

[19] Higo T, Hattori M, Nakamura T, Natsume T, Michikawa T, et al (2005) Subtype-specific and ER lumenal environment-dependent regulation of inositol 1,4,5-trisphosphate receptor type 1 by ERp44. Cell 120: 85–98. 15652484 10.1016/j.cell.2004.11.048

[20] Futatsugi A, Nakamura T, Yamada MK, Ebisui E, Nakamura K, et al (2005) IP_3_ receptor types 2 and 3 mediate exocrine secretion underlying energy metabolism. Science 309: 2232–2234. 16195467 10.1126/science.1114110

[21] Matsumoto M, Nakagawa T, Inoue T, Nagata E, Tanaka K, et al (1996) Ataxia and epileptic seizures in mice lacking type 1 inositol 1,4,5-trisphosphate receptor. Nature 379: 168–171. 8538767 10.1038/379168a0

[22] Potter BVL, Lampe D (1995) Chemistry of inositol lipid mediated cellular signaling. Angew Chem Int Ed Eng 34: 1933–1972.

[23] Sureshan KM, Riley AM, Rossi AM, Tovey SC, Dedos SG, et al. (2009) Activation of IP_3_ receptors by synthetic bisphosphate ligands. Chem Comm: 1204–1206. 10.1039/b819328b PMC2898634 19240874

[24] Mills SJ, Potter BVL (1996) Synthesis of d- and l-*myo*-inositol 1,4,6-trisphosphate, regioisomers of a ubiquitous second messenger. J Org Chem 61: 8980–8987. 11667881 10.1021/jo961280x

[25] Inoue T, Kikuchi K, Hirose K, Iino M, Nagano T (1999) Synthesis and evaluation of 1-position-modified inositol 1,4,5-trisphosphate analogs. Bioorg Med Chem Lett 9: 1967–1702. 10.1016/s0960-894x(99)00256-5 10397504

[26] Sureshan KM, Riley AM, Thomas MP, Tovey SC, Taylor CW, et al (2012) Contribution of phosphates and adenine to the potency of adenophostins at the IP_3_ receptor: synthesis of all possible bisphosphates of adenophostin A. J Med Chem. 55: 1706–1720. 10.1021/jm201571p PMC3285137 22248345

[27] Poinas A, Backers K, Riley AM, Mills SJ, Moreau C, et al (2005) Study of the interaction of the catalytic domain of Ins(1,4,5)P_3_ 3-kinase A with inositol phosphate analogues. ChemBioChem 6: 1449–1457. 15997461 10.1002/cbic.200400443

[28] Riley AM, Mahon MF, Potter BVL (1997) Rapid synthesis of the enantiomers of *myo*-inositol-1,3,4,5-tetrakisphosphate by direct chiral desymmetrization of *myo*-inositol orthoformate. Angew Chem Int Ed Eng 36: 1472–1474.

[29] Cardy TJA, Traynor D, Taylor CW (1997) Differential regulation of types 1 and 3 inositol trisphosphate receptors by cytosolic Ca^2+^ . Biochem J 328: 785–793. 9396721 10.1042/bj3280785 PMC1218987

[30] Rossi A, Sureshan KM, Riley AM, Potter BVL, Taylor CW (2010) Selective determinants of inositol 1,4,5-trisphosphate and adenophostin A interactions with type 1 inositol 1,4,5-trisphosphate receptors. Br J Pharmacol 161: 1070–1085. 20977457 10.1111/j.1476-5381.2010.00947.x PMC2998688

[31] Tovey SC, Dedos SG, Rahman T, Taylor EJA, Pantazaka E, et al (2010) Regulation of inositol 1,4,5-trisphosphate receptors by cAMP independent of cAMP-dependent protein kinase. J Biol Chem 285: 12979–12989. 20189985 10.1074/jbc.M109.096016 PMC2857138

[32] Sugawara H, Kurosaki M, Takata M, Kurosaki T (1997) Genetic evidence for involvement of type 1, type 2 and type 3 inositol 1,4,5-trisphosphate receptors in signal transduction through the B-cell antigen receptor. EMBO J 16: 3078–3088. 9214625 10.1093/emboj/16.11.3078 PMC1169926

[33] Pantazaka E, Taylor CW (2011) Differential distribution, clustering and lateral diffusion of subtypes of inositol 1,4,5-trisphosphate receptor. J Biol Chem 286: 23378–23387. 21550988 10.1074/jbc.M111.236372 PMC3123102

[34] Rahman TU, Skupin A, Falcke M, Taylor CW (2009) Clustering of IP_3_ receptors by IP_3_ retunes their regulation by IP_3_ and Ca^2+^ . Nature 458: 655–659. 19348050 10.1038/nature07763 PMC2702691

[35] Tovey SC, Sun Y, Taylor CW (2006) Rapid functional assays of intracellular Ca^2+^ channels. Nature Prot 1: 259–263. 10.1038/nprot.2006.40 17406242

[36] Eswar N, Webb B, Marti-Renom MA, Madhusudhan MS, Eramian D, et al (2006) Comparative protein structure modeling using Modeller. Curr Prot Bioinform 15: 5.6.1–5.6.30. 10.1002/0471250953.bi0506s15 PMC4186674 18428767

[37] Davis IW, Leaver-Fay A, Chen VB, Block JN, Kapral GJ, et al (2007) MolProbity: all-atom contacts and structure validation for proteins and nucleic acids. Nucleic Acids Res 35: W375–W383. 17452350 10.1093/nar/gkm216 PMC1933162

[38] Pettersen EF, Goddard TD, Huang CC, Couch GS, Greenblatt DM, et al (2004) UCSF Chimera–a visualization system for exploratory research and analysis. J Comput Chem 25: 1605–1612. 15264254 10.1002/jcc.20084

[39] Trott O, Olson AJ (2010) AutoDock Vina: improving the speed and accuracy of docking with a new scoring function, efficient optimization, and multithreading. J Comput Chem 31: 455–461. 19499576 10.1002/jcc.21334 PMC3041641

[40] Merlino A, Benitez D, Campillo NE, Paez JA, Tinoco LW, et al (2012) Amidines bearing benzofuroxan or benzimidazole 1,3-dioxide core scaffolds as *Trypanosoma cruzi-*inhibitors: structural basis for their interactions with cruzipain. Med Chem Comm 3: 90–101.

[41] Irvine RF, Schell MJ (2001) Back in the water: the return of the inositol phosphates. Nat Rev Mol Cell Biol 2: 327–338. 11331907 10.1038/35073015

[42] Loomis-Husselbee JW, Walker CD, Bottomley JR, Cullen PJ, Irvine RF, et al (1998) Modulation of Ins(2,4,5)*P* _3_-stimulated Ca^2+^ mobilization by Ins(1,3,4,5)*P* _4_: enhancement by activated G-proteins, and evidence for the involvement of a GAP1 protein, a putative Ins(1,3,4,5)*P* _4_ receptor. Biochem J 331: 947–952. 9560326 10.1042/bj3310947 PMC1219439

[43] Bird GSJ, Putney JW Jr (1996) Effect of inositol 1,3,4,5-trisphosphate on inositol trisphosphate-activated Ca^2+^ signaling in mouse lacrimal cells. J Biol Chem 271: 6766–6770. 8636098 10.1074/jbc.271.12.6766

[44] Burgess GM, McKinney JS, Irvine RF, Putney JW (1985) Inositol 1,4,5-trisphosphate and inositol 1,3,4-trisphosphate formation in Ca^2+^-mobilizing-hormone-activated cells. Biochem J 232: 237–243. 3002326 10.1042/bj2320237 PMC1152864

[45] Wilcox RA, Primrose WU, Nahorski SR, Challiss RAJ (1998) New developments in the molecular pharmacology of the *myo*-inositol 1,4,5-trisphosphate receptor. Trends Pharmacol Sci 19: 467–475. 9850611 10.1016/s0165-6147(98)01260-7

[46] Nerou EP, Riley AM, Potter BVL, Taylor CW (2001) Selective recognition of inositol phosphates by subtypes of inositol trisphosphate receptor. Biochem J 355: 59–69. 11256949 10.1042/0264-6021:3550059 PMC1221712

[47] Kozikowski AP, Ognyanov VI, Fauq AH, Nahorski SR, Wilcox RA (1993) Synthesis of 1d-3-deoxy-, 1d-2,3-dideoxy, and 1d-2,3,6-trideoxy-*myo*-inositol 1,4,5-trisphosphate from quebrachitol, their binding affinities, and calcium release activity. J Am Chem Soc 115: 4429–4434.

[48] Inoue T, Kikuchi K, Hirose K, Iino M, Nagano T (2001) Small molecule-based laser inactivation of inositol 1,4,5-trisphosphate receptor. Chem Biol 8: 9–15. 11182315 10.1016/s1074-5521(00)00051-x

[49] Hagiwara T, Motomizu S (1994) Equilibrium and kinetic studies on the formation of the triphenylmethanols from triphenylmethane dyes. Bull Chem Soc Jpn 67: 390–397.

[50] Bosanac I, Yamazaki H, Matsu-ura T, Michikawa M, Mikoshiba K, et al (2005) Crystal structure of the ligand binding suppressor domain of type 1 inositol 1,4,5-trisphosphate receptor. Mol Cell 17: 193–203. 15664189 10.1016/j.molcel.2004.11.047

[51] Lin CC, Baek K, Lu Z (2011) Apo and InsP_3_-bound crystal structures of the ligand-binding domain of an InsP_3_ receptor. Nat Struct Mol Biol 18: 1172–1174. 21892169 10.1038/nsmb.2112 PMC3242432

[52] DeLisle S, Radenberg T, Wintermantel MR, Tietz C, Parys JB, et al (1994) Second messenger specificity of the inositol trisphosphate receptor: reappraisal based on novel inositol phosphates. Am J Physiol 266: C429–C436. 8141257 10.1152/ajpcell.1994.266.2.C429

[53] Lu P-J, Gou D-M, Shieh W-R, Chen C-S (1994) Molecular interactions of endogenous d-*myo*-inositol phosphates with the intracellular d-*myo*-inositol 1,4,5-trisphosphate recognition site. Biochemistry 33: 11586–11597. 7918372 10.1021/bi00204a021

[54] Nakanishi W, Kikuchi K, Inoue T, Hirose K, Iino M, et al (2002) Hydrophobic modifications at 1-phosphate of inositol 1,4,5-trisphosphate analogues enhance receptor binding. Bioorg Med Chem Lett 12: 911–913. 11958992 10.1016/s0960-894x(02)00044-6

[55] Wilcox RA, Fauq A, Kozikowski AP, Nahorski SR (1997) Defining the minimal structural requirements for partial agonism at the type I *myo*-inositol 1,4,5-trisphosphate receptor. FEBS Lett 402: 241–245. 9037203 10.1016/s0014-5793(96)01540-2

[56] Marchant JS, Chang Y-T, Chung S-K, Irvine RF, Taylor CW (1997) Rapid kinetic measurements of ^45^Ca^2+^ mobilization reveal that Ins(2,4,5)*P* _3_ is a partial agonist of hepatic Ins*P* _3_ receptors. Biochem J 321: 573–576. 9032438 10.1042/bj3210573 PMC1218107

[57] Lampe D, Mills SJ, Potter BVL (1992) Total synthesis of the second messenger analogue d-*myo*-inositol 1-phosphorothioate,4,5-bisphosphate: optical resolution of dl-1-*O*-allyl-2,3,6-tri-O-benzyl-*myo*-inositol and fluorescent labelling of *myo*-inositol 1,4,5-trisphosphate. J Chem Soc Perkin Trans: 2899–2906.

